# HPV Prevalence in Multiple Anatomical Sites among Men Who Have Sex with Men in Peru

**DOI:** 10.1371/journal.pone.0139524

**Published:** 2015-10-05

**Authors:** Magaly M. Blas, Brandon Brown, Luis Menacho, Isaac E. Alva, Alfonso Silva-Santisteban, Cesar Carcamo

**Affiliations:** 1 Epidemiology, STD and HIV Unit, School of Public Health and Administration, Universidad Peruana Cayetano Heredia, Lima, Peru; 2 Center for Healthy Communities, UCR School of Medicine, Riverside, California, United States of America; 3 Unit of Health, Sexuality and Human Development, School of Public Health and Administration, Universidad Peruana Cayetano Heredia, Lima, Peru; State University of Maringá/Universidade Estadual de Maringá, BRAZIL

## Abstract

**Background:**

Human Papilloma Virus (HPV) infection is the most common sexually transmitted viral infection worldwide. HPV is highly prevalent in sexually active men who have sex with men (MSM) and has been associated with anal cancer, penile cancer, and oropharyngeal cancer.

**Methods:**

From March to September 2011, we conducted a cross-sectional study of HPV prevalence among MSM above age 18 years. Participants were recruited using respondent driven sampling at Clinica Cayetano Heredia. All participants provided anal, genital, and oral samples for HPV DNA testing, and blood for HIV and HPV antibody testing.

**Results:**

A total of 200 MSM were recruited in the study. The mean age was 34 years (range 18–59 years, SD = 9.4) and101 participants were HIV negative (99 HIV positive). HPV 6/11/16/18 or quadrivalent HPV vaccine (HPV4) genotype seroprevalence among HIV negative and positive MSM was 64.3% (55%-75.9%) and 93.8% (87.6%-99.2%) respectively (p<0.001). HIV positivity was associated with a higher prevalence of HPV4 and HPV 16/18 DNA at external genital sites and the anal canal. HPV4 DNA prevalence at external genital sites among HIV negative and positive MSM was 14.9% and 28.7% (p = 0.02) respectively, at anal canal was 50.9% and 79.0% (p = 0.001), and at the oral cavity was 9.9% and 8.5% (p = 0.6).

**Conclusions:**

HPV4 seroprevalence was high in our study among both HIV positives and negatives, with HPV DNA prevalence much lower, and the anal canal being the anatomical site with the highest HPV DNA prevalence. HPV prevention interventions are needed among MSM at high-risk for HIV infection.

## Introduction

HPV infection is the most common sexually transmitted viral infection worldwide. Viral sexually transmitted infections (STIs) account for 94% of the total economic burden of STIs, with the Human Papilloma Virus (HPV) among the most costly [1]. HPV is highly prevalent in sexually active men and has been associated with 80–85% of anal cancers and 50% of penile cancers. It has also been associated with oropharyngeal cancers and laryngeal cancers [2–6]. HPV seroprevalence is approximately 2 to 6 times higher among men who have sex with men (MSM) and among men who have sex with men and women (MSMW) compared to heterosexual men [7].

In general, studies show HPV prevalence varies by anatomical site (penis, anus, mouth/throat) and is higher among HIV positive MSM compared to their HIV negative counterparts [8]. Moreover, the incidence of invasive anal cancer among HIV-positive MSM is approximately twice that of HIV-negative MSM [9].

Although studies have generated significant HPV data among MSM from US and Europe, there is limited data about HPV prevalence among South American MSM [8–16]. Better understanding of the HPV burden on MSM will help advocate for routine anal screening in this population, as well as identify priorities for HPV vaccination campaigns. The primary objectives of this study were to ascertain the type-specific prevalence of HPV types at different anatomical sites (anus, penis, mouth/throat) and HPV antibody in blood among a sample of MSM in Peru.

## Materials and Methods

We received ethical approval from the Institutional Review Board of Universidad Peruana Cayetano Heredia in Lima, Peru. All enrollees provided a written informed consent prior to their participation in our study.

### Study Design and Recruitment

Between March and September 2011, we conducted a cross-sectional study of HPV prevalence among MSM in Lima, Peru who were 18 years and older. Participants were recruited through respondent driven sampling (RDS) starting from Clinica Cayetano Heredia. RDS is a type of chain-referral sampling that is used to recruit hidden populations, such as MSM. Contrary to the snowball sampling, RDS yields a sample that is independent of the initial subjects (the seeds), regardless whether they are recruited randomly or not. In this study, each subject could recruit up to three participants to ensure a wide and varied selection of subjects, and to avoid that few subjects recruit the whole sample.

Seeds received an explanation of the study, and three coupons with unique serial numbers that were used to recruit a peer who was eligible for the study. If the peer enrolled in the study, the seed was eligible for an incentive for the recruitment effort that included 20 condoms for the first and second recruit and one backpack valued at $8 US Dollars for the third recruit. Each referred respondent received 3 coupons, until the desired sample size was met. No attempt was made to recruit participants by HIV status.

The inclusion criteria for the study included: 1) Being a male 18 years of age or older, 2) self-reported anal sex with another man within 12 months prior to enrollment, 3) live in Lima, 4) Willing to provide informed consent for the collection of general demographic and sex behavior data, as well as blood, anal, genital, and oral samples for HPV and HIV testing, and anal Pap smear. The exclusion criteria for the study included: 1) Prior participation in an HPV vaccine clinical trial and, 2) History of anal or penile cancer.

The interviewer administered a cell-phone based survey to participants that included seven sections and 88 questions about demographic characteristics, sexual behavior, STI history, previous HIV testing, smoking status, drug and alcohol use, knowledge about HPV, HPV vaccination and HPV-related cancers, and willingness to receive the HPV vaccine. The survey also included questions about social network. We pilot tested the survey for understanding of language and accurate interpretation of questions prior to data collection.

Survey information was collected using EpiSurveyor Mobile (now known as MagPi), a mobile phone based application for collecting data in field-based surveys [17]. With this application the interviewer was able to fill the survey with a point-and-click interface. Some elements of the survey included check boxes, numeric-entry boxes, and selection lists to facilitate data entry. Once the data was collected in the cell phone, study personnel downloaded the information via WiFi directly to the database.

### Study Procedures and Laboratory Techniques

Following informed consent and completion of the survey, all participants underwent study procedures in the following order: 1) an oral rinse with Scope (Procter & Gamble, Cincinnati Ohio) to measure HPV in the oral cavity, 2) external genital lesion inspection by medical personnel and swab collection of the lesions using a magnifying glass, 3) collection of swab specimens separately from the glans, coronal sulcus, penis shaft and scrotum, 4) inspection of the perineal/perianal region, 5) collection of swab specimens from the anal canal 6) anal Papanicolaou 7) collection of anal swabs for Chlamydia and gonorrhea testing, and finally 8) blood collection for HPV and HIV antibody testing. All swabs were placed in a container with phosphate-buffered saline (PBS). The swabs from the glans, coronal sulcus, penis shaft and scrotum were combined into one sample.

HPV testing was performed at Johns Hopkins Bloomberg School of Public Health. Linear array testing was used to detect high-risk (16, 18, 26, 31, 33, 35, 39, 45, 51, 52, 53, 56, 58, 59, 66, 67, 68, 73, 82 and 82var) and low-risk (6, 11, 40, 42, 54, 55, 61, 62, 64, 69, 70, 71, 72, 81, 83, 84, and 89) HPV types (Roche Molecular Systems). For HPV DNA preparation, samples were digested with 20 μg/mL proteinase K for 1–2 h at 37°C. For each sample, 2400 μL was used to isolate DNA following the manufacturer's protocol (QIAamp DNA Blood Mini Kit, Qiagen Sciences, Gaithersburg, MD). Then, each DNA sample was amplified using HPV L1 consensus primers MY09, MY11, and HMB01 and β-globin primers PC04 and GH20. Five μl of PCR products were then dotted onto nylon filters and probed with biotin-labeled β-globin and HPV generic probes. Samples negative for β-globin were considered insufficient for HPV DNA testing.

Anal Pap slides were read by a cytopathologist at Cayetano Heredia National Hospital and rated according to the Bethesda 2001 criteria. We used a fourth-generation enzyme immunoassay (EIA) to detect HIV–1/2 IgG and IgM antibodies or HIV–1 p24 antigen (Genscreen ULTRA HIV Ag–Ab, Bio-Rad). Positive samples were confirmed by Western blot. HPV antibody testing for HPV types 6, 11, 16, and 18 was performed using competitive Luminex-based immunoassays (cLIA; developed by Merck Research Laboratories, West Point, PA, using technology from the Luminex Corporation, Austin, TX). Serostatus cutoffs were 20 mMU/mL for HPV 6, 16 mMU/mL for HPV 11, 20 mMU/mL for HPV 16, and 24 mMU/mL for HPV 18 [18].

### Statistical Methods

Point estimates and 95% confident intervals (95% CI) were calculated for variables of interest using Respondent Driven Sampling Analysis Tool 6.0.1 (RDSAT) (www.respondentdrivensampling.org).Estimates were generated by the software by adjusting for participants’ networks sizes and differential recruitment patterns. Demographic, behavioral and biological outcome variables were analyzed stratifying the study population according to their HIV status. RDSAT generated weights for the variable outcome (any HPV infection by HIV status) were exported and used for bivariate analysis as suggested in the literature [19–22]. That is, the outcome of interest was analyzed in RDSAT and then individualized weights were exported and merged with the dataset. Further analyses (chi2 to test differences by HIV status) were weighted using this weight, using STATA 10.0 (College Station, TX).

## Results

**Fig 1:**
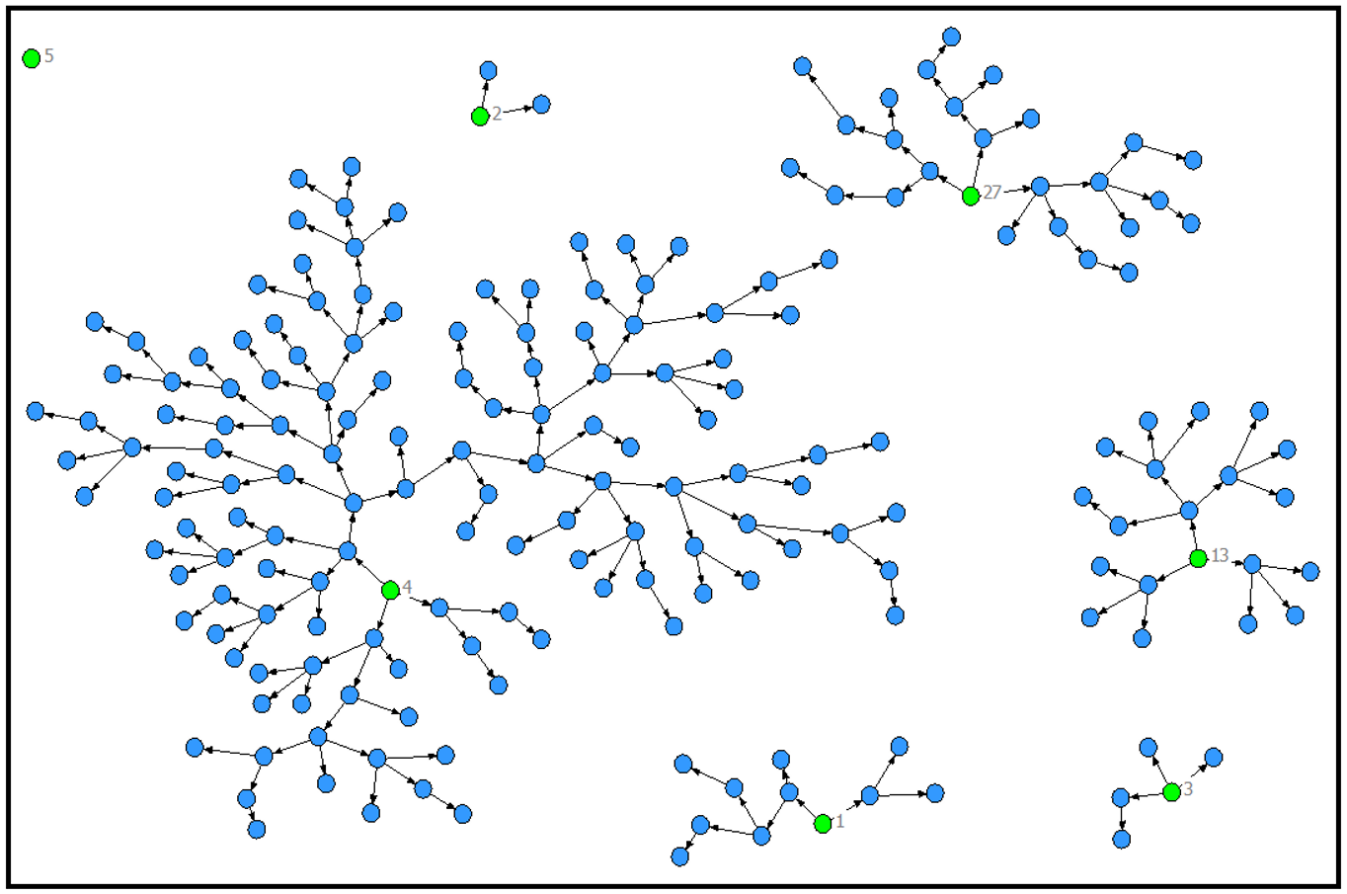
Representation of the seeds and their referred participants.

A total of 200 MSM were enrolled in our study. Initially, we recruited a total of five participants (three MSM, one MSMW and one transgender woman) who served as “seeds” to help identify other target group members. Two of the seeds did not refer the required three participants, and thus we recruited two additional seeds (one MSM and one MSMW). Fig 1 illustrates the seeds and their referred participants.

In total, 101 (50.5%) HIV negative and 99 (49.5%) HIV positive MSM were recruited. The mean age of participants was 34 years (minimum: 18 and maximum: 59, standard deviation 9.4). The demographic characteristics of the participants are shown in Table 1. The majority of our participants self-identified as gay and had mostly sex with other men. The most commons STI symptoms were dysuria, anal bleeding and anal pain. Regarding self-reported STI symptoms during their current visit, 4.4% of HIV positive MSM and 4.1% of HIV negative MSM reported genital warts (p = 0.8), and 11% of HIV positive MSM and 6.6% of HIV negative MSM reported anal warts (p = 0.5). Significantly more HIV positive participants used a condom at last anal intercourse (p<0.001).

**Table 1:** Demographic characteristics and sexual history among HIV positive and negative men who have sex with men.

Subject characteristic	HIV Positive (N = 99)	HIV Negative (N = 101)	
	N (%)	N (%)	p-value
**Age (years)**			0.002
18–20	0 (0)	11 (10.9)	
21–24	6 (6.1)	17 (16.8)	
25–35	36.3	46 (45.5)	
>35	57 (57.6)	27 (26.8)	
**Education**			0.12
<High school	3 (5.9)	2 (0.7)	
High school graduate	40 (35.9)	46 (61.4)	
University/Technical non-graduate	27 (31.0)	22 (16.0)	
University/Technical graduate	29 (27.2)	31 (21.9)	
**Belongs to an indigenous group**	1 (1.0)	2 (1.9)	0.83
**Ever smoked cigarettes**	98 (99.7)	97 (95)	<0.001
**Current smoker**	42 (44.2)	49 (50.1)	0.12
**Had sex under the influence of alcohol within the last month**	18 (9.7)	31 (29.1)	0.05
**Had sex under the influence of a drug within the last month**	2 (1.8)	3 (4.9)	0.02
**Sexual self-perception**			0.08
Gay	79 (79.8)	55 (54.5)	
Bisexual	11 (11.1)	22 (21.8)	
Trans^&^	4 (4.1)	13 (12.9)	
*Hombre* (man)	4 (4.1)	7 (6.9)	
Woman	1 (1)	4 (3.9)	
**Sexual orientation**			
Homosexual	56 (58.1)	61 (58.2)	0.35
Bisexual	43 (41.9)	40 (41.8)	
**Sexual role**			
Insertive	3 (3.8)	13 (15.6)	0.07
Receptive	12 (11.3)	14 (10.7)	
Versatile	84 (85.0)	74 (73.8)	
**Self-reported STIs symptoms during the last six months**			
Urethral discharge	1 (0.5)	5 (7.7)	<0.001
Dysuria	30 (33.7)	33 (34.6)	0.52
Genital ulcers	13 (12.9)	14 (12.2)	0.71
Anal discharge	6 (6.8)	4 (7.5)	0.91
Anal bleeding	32 (34.1)	46 (47.7)	0.06
Anal pain	37 (37.1)	31 (32.5)	0.89
Anal ulcers	22 (23.4)	26 (21.3)	0.71
**Ever had genital warts**	12 (13.7)	4 (4.1)	0.05
**STIs diagnosed by a health provider during the last six months**	14 (11.6)	17 (16.3)	0.66
**Ever had oral sex**	93 (95.7)	88 (82.8)	0.04
**Percentage of unprotected anal intercourse during the last 3 months**			
0–25	71 (84.9)	54 (52.6)	0.002
26–50	5 (4.5)	20 (22.6)	
51–75	3 (1.8)	1 (0.4)	
76–100	8 (8.8)	20 (24.4)	
**Used condom at last anal intercourse**	84 (89.5)	60 (56.7)	<0.0001
**Used condom at last oral sex**	27 (27.9)	19 (11.9)	0.01
**Ever had sexual intercourse with a woman within the last 12 months**	7 (5.9)	18 (22.5)	0.1
**Received money for sexual intercourse within the last six months**	12 (11.2)	27 (22.3)	

^&^Includes transvestite, transgender and transsexual

Overall HPV prevalence was significantly higher among HIV positives than negatives at the external genital sites (44.3% vs 22.4%, p <.01), in the anal canal (97.1% vs 73.3%, p<0.01), and in the oral cavity (44.3% vs 22.4%, p = 0.04). Seroprevalence of HPV 6/11/16/18 or quadrivalent HPV vaccine (HPV4) genotypes in both HIV negative (64.3%) and HIV positive (93.8%) MSM was high, and significantly higher among HIV positive MSM (p<0.001), as well as the HPV prevalence at the anal canal, followed by the prevalence at external genital sites and oral cavity. Similarly, the prevalence of high-risk HPV types at the anal canal was higher among HIV positive compared to HIV negative MSM (Table 2). When comparing HPV4 DNA and HPV4 serology prevalence, serology was significantly higher among all participants (p = 0.001) and for HIV negative participants (p = 0.002), but there was no significant difference in prevalence among HIV positive participants (p = 0.94).

**Table 2:** Summary of anogenital HPV DNA and seroprevalence among 99 HIV positive and 101 HIV negative men who have sex with men.

	HPV DNA positivity											
HPV Type	External Genital Sites			Anal Canal			Oral Cavity			Serum		
	HIV Status		P-Value	HIV Status		P-Value	HIV Status		P-Value	HIV Status		P-Value
	Negative N (%)	Positive N (%)		Negative N (%)	Positive N (%)		Negative N (%)	Positive N (%)		Negative N (%)	Positive N (%)	
**Any tested type** *	41 (22.4)	61 (44.3)	**0.004**	77 (73.3)	96 (97.1)	**<0.001**	26 (22.4)	40 (44.3)	**0.04**	NA	NA	
**Any high-risk type**	24 (27.9)	41 (38.5)	0.23	57 (55.6)	82 (86.0)	**<0.001**	15 (13.8)	27 (24.8)	0.23	NA	NA	
**HPV 6/11/16/18**	15 (14.9)	32 (28.7)	**0.02**	50 (50.9)	63 (79.0)	**0.001**	11 (9.9)	11 (8.5)	0.60	66 (64.3)	91 (93.8)	**<0.001**
**HPV 16/18**	8 (7.6)	16 (15.4)	**0.04**	28 (25.5)	35 (49.3)	**0.008**	9 (8)	11 (8.4)	0.85	36 (30.6)	56 (64.8)	**<0.001**
**HPV 16**	6 (6.5)	11 (11.9)	0.18	18 (13.4)	23 (33.9)	**0.01**	9 (8)	11 (8.4)	0.85	45 (28.5)	33 (50.0)	**0.02**
**HPV 18**	3 (1.9)	6 (3.7)	0.08	14 (18.1)	20 (31.7)	**0.05**	5 (2.4)	6 (4.2)	0.51	19 (10.7)	15 (19.3)	0.11
**HPV 6/11**	11 (11.3)	22 (21.8)	0.07	34 (31.5)	41 (47.5)	0.14	4 (2.4)	4 (3)	0.68	58 (54.9)	82 (82.8)	**0.004**
**HPV 6**	10 (9.9)	13 (14.5)	0.41	23 (20)	29 (37.3)	0.12	4 (2.5)	3 (2.1)	0.98	51 (48.6)	70 (75.7)	**0.004**
**HPV 11**	2 (1.8)	9 (7.3)	**0.05**	15 (13.7)	17 (16.7)	0.59	0 (0.4)	1 (1.4)	0.24	29 (25.8)	53 (50.3)	0.07
**HPV 31**	3 (2.2)	1 (1.2)	0.57	5 (3.6)	15 (14.1)	**0.007**	4 (3.5)	6 (5.8)	0.39	NA	NA	NA
**HPV 33**	2 (3)	3 (3.3)	0.84	6 (6.4)	8 (6.9)	0.80	1 (1.4)	2 (5.1)	**0.05**	NA	NA	NA
**HPV 45**	3 (3.5)	3 (6.5)	0.97	11 (12.1)	14 (15.2)	0.44	6 (4.9)	10 (7.7)	0.45			
**HPV 52**	3 (1.9)	5 (2.9)	0.65	11 (9)	17 (24.2)	0.08	4 (3.3)	4 (1.3)	0.66	NA	NA	NA
**HPV 58**	2 (1.9)	8 (7.9)	0.03	9 (9.8)	22 (21.1)	**0.04**	5 (4.5)	12 (9.6)	0.25	NA	NA	NA

NA = Not Available

*A total of 37 HPV types tested

## Discussion

We collected HPV DNA at three anatomical sites and HPV serology at the same point for each participant, allowing for cross-sectional associations. This is the first study in South America we are aware of that has evaluated prevalence at multiple anatomical sites among HIV positive and negative MSM. We found a high exposure to the HPV4 vaccine types. In terms of HPV DNA positivity, HPV prevalence at the anal canal was higher than at external genital sites and oral cavity. Other studies have also reported higher prevalence at the anal canal, the proposed reason is the susceptibility of the anal skin and the frequent trauma caused during anal sex [11].

Compared to the national surveillance and studies having used RDS in Latin America, our sample has much higher rates of HIV. For example, the prevalence of HIV in studies in Argentina and Brazil was 17.3% and 7.6%, respectively, compared to 49.5% in our study [23, 24]. The prevalence of HIV in Peru reported by the national surveillance is 12.4%, far less than in our study [25]. Our project successfully implemented RDS and attracted a cohort of HIV infected MSM interested in receiving medical care, which may have not been reached by national surveillance studies.

HIV positivity was associated with a higher prevalence of HPV of any type at external genital sites, anal canal and oral cavity. HIV positivity was also associated with a higher prevalence of HPV4 and HPV 16/18 at external genital sites and anal canal. Several studies have found higher HPV prevalence among HIV positive MSM compared to HIV negative MSM [8,11]. This higher HPV prevalence in HIV positive participants may be due to an increased persistence of HPV infection due to compromised immunity or to a high incidence of new HPV infections as a consequence of sexual behavior [26].

A lower HPV prevalence among those with a negative HIV status is expected [27]. At external genital sites, high-risk HPV prevalence was 27.9% for HIV negative MSM and 38.5% for HIV positive MSM in our study. Similarly, a study in the Netherlands found that the prevalence of penile high-risk HPV infection was lower in HIV negative MSM compared to HIV positive (16% vs 32%) MSM [11]. The penile prevalence in Netherlands was much lower than our pooled external genital site prevalence which included penile HPV. Additionally, in our study, at the anal canal, the HPV prevalence of high-risk HPV types was 55.6% for HIV negative MSM and 86.0% for HIV positive MSM. Similarly, a study in the United States found that the oncogenic HPV prevalence was higher among HIV- infected (61.3 vs 39.7%) than uninfected MSM [28]. At the oral cavity, the HPV prevalence of high-risk HPV types was 14% for HIV negative MSM and 25% for HIV positive MSM, with HPV–16 being the most common high-risk oral HPV type in our study, with an 8% prevalence. Additional studies have also found higher HPV prevalence among HIV positive than negative MSM in the oral cavity. Another study in the Netherlands detected oncogenic HPV types in 24.8% of HIV positive and 8.8% of HIV negative MSM. Of these high-risk types, HPV–16 was also the most common [29]. While oral HPV prevalence is lower than external genital HPV prevalence among MSM, it is still higher than that in the general population in Peru, pointing to a potential need for oral cancer screening [30].

Our study has some limitations. The 200 MSM included in the study may not be representative of the general MSM population in Lima. Also, subjects recruited depended on the participant seeds, which likely recruited participants who were similar to them and in their network. In addition, the cross sectional nature of this study precludes the determination of the temporal relationship between HIV and HPV.

HPV4 seroprevalence was high in our study, with nearly all participants with HIV having HPV4 serotypes. HPV4 DNA prevalence was much lower, illustrating that HPV4 DNA is not a good measurement of HPV exposure. Although some Latin American governments, including Peru, now provide HPV vaccination to girls, advocacy is needed to include groups who may be at higher risk of HPV associated disease such as MSM.
